# The nature and epidemiology of OqxAB, a multidrug efflux pump

**DOI:** 10.1186/s13756-019-0489-3

**Published:** 2019-02-22

**Authors:** Jun Li, Heying Zhang, Jianan Ning, Abdul Sajid, Guyue Cheng, Zonghui Yuan, Haihong Hao

**Affiliations:** 1 National Reference Laboratory of Veterinary Drug Residues (HZAU) and Key Laboratory of the Detection for Veterinary Drug Residues, Wuhan, 430070 Hubei China; 2 Institute of Veterinary Medicine, Jiangsu Academy of Agricultural Sciences, Nanjing, 210014 Jiangsu China; 3 Laboratory of Quality & Safety Risk Assessment for Livestock and Poultry Products (Wuhan), Ministry of Agriculture, P.R China, Wuhan, 430070 Hubei China; 4 College of Veterinary Sciences and Animal Husbandry, Abdul Wali Khan University Mardan, Mardan, KP Pakistan

**Keywords:** *oqxAB*, Multidrug efflux pump, Quinoxalines, Quinolones, Tigecycline, Nitrofurantoin

## Abstract

**Background:**

OqxAB efflux pump has been found to mediate multidrug resistance (MDR) in various bacteria over the past decades. The updates on the nature and epidemiology of OqxAB efflux pump need to be fully reviewed to broaden our understanding of this MDR determinant.

**Methods:**

A literature search using the keyword of “oqxAB” was conducted in the online databases of Pubmed and ISI Web of Science with no restriction on the date of publication. The 87 publications were included into this review as references due to their close relevance to the nature and/or epidemiology of OqxAB efflux pump.

**Results:**

The *oqxAB* gene generally locates on chromosome and/or plasmids flanked by IS26-like elements in clinical isolates of *Enterobacteriaceae* and *Klebsiella pneumoniae*, conferring low to intermediated resistance to quinoxalines, quinolones tigecycline, nitrofurantoin, several detergents and disinfectants (benzalkonium chloride, triclosan and SDS). It could co-spread with other antimicrobial resistance genes (*bla*_CTX-M_, *rmtB* and *aac(6′)-Ib* etc.), virulence genes and heavy metal resistance genes (*pco* and *sil* operons). Both RarA (activator) and OqxR (repressor) play important roles on regulation of the expression of OqxAB.

**Conclusions:**

The dissemination of *oqxAB* gene may pose a great risk on food safety and public health. Further investigation and understanding of the natural functions, horizontal transfer, and regulation mechanism of the OqxAB efflux pump will aid in future strategies of antimicrobial usage.

## Background

Antimicrobial resistance has posed increasing challenge to public health [1]. Efflux pumps are found in almost all bacterial species and have important roles on both intrinsic and acquired resistance to antimicrobials by lowering intracellular antibiotic concentration and promoting site mutation accumulation [2, 3]. Most of the efflux pumps are located on the chromosome of bacteria [4]. Plasmid-mediated efflux pumps have been described in recent years, such as QacBIII [5], Tet(L) [3] and MexCD [6] efflux pumps.

In 2004, a novel plasmid-encoded multidrug efflux pump OqxAB was firstly identified on the pOLA52 plasmid in *E. coli* from swine manure in Denmark [7]. The prevalence of *oqxAB* among *Enterobacteriaceae* have been increasingly reported over the past decades (Tables 1, 2 and 3) [8]. The overexpression of OqxAB confers resistance to multiple drugs (quinoxalines, quinolones, tigecycline, nitrofurantoin and chloramphenicol), detergents and disinfectants (benzalkonium chloride, triclosan and SDS). This plasmid-borne multidrug efflux pump may pose a resistance problem, because it could facilitate the development of resistance to multiple drugs and dissemination of antimicrobial resistance via horizontal transfer.

**Table 1:** Epidemiology of *oqxAB* in *in Escherichia coli* and *Enterobacter cloacae*

References	Year(s) of isolates collection	Geographic area	Sample source	Percentage of *oqxAB* carring isolates (no. of isolates)	Resistance phenotype(s)	Descriptions
[27]	1995–1998	Danmark, Swedish	Swine	11.84% (27/228)	OLA	*oqxAB* operon was situated on pOLA52 plasmid in most strains.
[17]	2002	Guangdong, China	Swine, chicken, environment, farmworker	Animals: *E. coli*: 39.8% (39/98), environment: *E. coli*:43.9% (18/41), farmworkers: *E. coli*:30.3% (10/33)	MEQ, OLA, CHL, ENR, CIP, AMP, KAN, TET, SXT	*oqxAB* was associated with IS26 and was carried on the 43- to 115-kb IncF transferable plasmid.
[31]	2012–2014	Zhengzhou, China	Dog, cats, human	Dogs: *E. coli*:58.5% (62/106), cats: *E. coli*:56.25% (36/64), human: *E. coli*:42.0% (42/100)	OLA, MEQ, CIP, TET, FFC	Several *oqxAB*-positive isolates have high similarity and the *oqxAB* gene was primarily located on plasmids.
[32]	1970s-2013	North and South China	Chicken, pig, duck, goose	*E. coli*:28.7% (322/1123)	MEQ	IS26-flanked Tn*6010* element was prone to excision via IS26-mediated recombination.
[40]	2011–2013	Guangdong, China	Pig, chicken, retail meat, humans	Animals: *E. coli*: 33.8% (172/509), food: *E. coli*:17.3% (60/346), human: *E. coli*:18.1% (90/498)	NEO, APR, FFC, OLA, TET, SXT	*oqxAB* was located on plasmids belonging to IncN1-F33:A-:B-, IncHI2/ST3, F-:A18:B-, F-:A-:B54
[41]	2010–2011	China	Human	*E. coli*:3.8% (23/590)	CIP, LVX	*oqxB20* and *oqxB29* were identified.
[43]	2001–2015	Taiwan, China	Human	*E. coli*:6.05% (15/248)	CIP, LVX	*oqxAB* was located on plasmid and successfully transferred to *E. coli* C600 by conjugation.
[44]	2008–2010	Ujjain, India	Hospital wastewater	*E. coli*:1.05% (2/190)	CTX, CAZ, CIP, OFX	First paper reports the detection of *oqxAB*-carring *E.coli* from hospital wastewater in India
[45]	1993–2010	China	Human, animals, environment	Human: *E. coli*:5.2% (16/307), chicken: *E. coli*:19.8% (76/384), pigs: *E. coli*:51% (101/198), environment: *E. coli*: 20.5% (9/44)	AMP, TET, SXT, CHL, STR, CIP	First report of *oqxAB*-positive isolates from ducks and geese and as early as 1994 from chickens.
[48]	2004–2011	Guangdong, Anhui, Guangxi, Henan, Jiangsu, Sichuan, Fujian, Jiangxi, Beijing, China	Pigs, chickens and ducks), companion animals, human, retail meat	Pig: *E. coli*:55.7% (280/503), chicken: *E. coli*:25.8% (127/493), duck: *E. coli*:40.6% (109/389), pet: *E. coli*:10% (35/353), food: *E. coli*: 16.2% (57/352), human: *E. coli*:7.2% (15/207)	CIP	43.6% of the *E. coli* harbored at least one PMQR gene. The most common PMQR gene was *oqxAB* (29.3%), followed by *qnr* (13.6%), *aac(6′)-Ib-cr* (11.6%), and *qepA* (3.3%).
[49]	2002–2010	Guangdong, China	Ducks, chickens, pigs	*E. coli*:43.43% (215/495)	CIP, ENR, LVX, NAL	Prevalence of *oqxAB* had significant Spearman correlation coefficients with MICs of quinolones.
[50]	2011	Beijing, Shanxi, Guangdong, Inner Mogolia, China	Chicken carcasses, ground pork	*E. coli*:62.26% (66/106)	CTX, CIP,CHL, TET, GEN, SXT, AMP, CAZ	68.2% of the cefotaxime and ciprofloxacin coresistant *E. coli* isolates carried at least one PMQR gene and eight subtypes of *bla*_CTX-M_ were identified.
[46]	2006–2008	Northern Italy	Farm and wild lagomorphs	*E. coli*:15% (17/113)	TET, STR, NAL, SXT, CHL	Seven out of 17 strains were carried from three to six different plasmid types, such as IncF, IncHI1, IncI1, IncN, IncP, IncX1, IncY, and ColE.
[16]	2002–2012	Guangdong, China	Duck, chicken, geese and pig	*E. coli*:47.12% (328/696)	AMP, CHL,FFC, TET, GEN, KAN, CTI, DOX	*oqxAB*, *bla*_CTX-M_ and *floR* were co-transferred on F33:A-: B- and HI2 plasmids in *E. coli.*
[19]	1998–2006	Seoul Korea	Human	*E. coli*:0.4% (1/261) *E. cloacae*: 4.6% (3/65),	OLA, CIP	This is the first report of the presence of an *oqxAB*-containing plasmid in a human isolate of *E. coli*.
[14]	2010	Shanghai China	Human	*E. coli*: 6.6% (9/136),	NAL, CIP, NOR, LVX, OLA, TMP, CHL, TET	Variants of *oqxA2*, *oqxB2* and *oqxB3* were identified in two *E. coli* strains.

Notes: *OLA* olaquindox, *MEQ* mequindox, *CHL* chloramphenicol, *ENR* enrofloxacin, *CIP* ciprofloxacin, *AMP* ampicillin, *KAN* kanamycin, *TET* tetracycline, *SXT* sulfamethoxazole-trimethoprim, *FFC* florfenicol, *NEO* neomycin, *APR* apramycin, *LVX* levofloxacin, *CTX* cefotaxime, *CAZ* ceftazidime, *OFX* ofloxacin, *STR* streptomycin, *GEN* gentamicin, *CTI* ceftiofur, *DOX* doxycycline, *CTX* cefotaxime, *NAL* nalidixic acid

**Table 2:** Epidemiology of *oqxAB* in *Salmonella* spp.

References	Year(s) of isolates collection	Geographic area	Sample source	Percentage of *oqxAB* carring isolates (no. of isolates)	Resistance phenotypes	Descriptions
[58]		Hong Kong	Pork, chicken	2.38 (2/84)	TET, CHL, NAL, OLA	First Detection of *oqxAB* on the chromosomes of two *Salmonella* Derby isolates from food.
[60]	2005–2011	Guangdong, Guangxi, Henan, Fujian, Sichuan, Beijing, Shanghai, Chongqing, China	Human	0.73% (4/546)	NAL, CHL, GEN, KAN, TET	Combined effects of ESBL determinants and *oqxAB* were responsible for the emergence of XDR *S*. Typhimurium.
[59]	2009–2013	Henan, China	Poultry, human	72.73% (112/154)	AMP, CAZ, CHL, CIP, CTX, GEN, SXT, TET	Co-occurrence of *qepA*, *oqxAB*, and *aac(6′)-Ib-cr* with mutations in *gyrA* and *parC* and several ESBLs were noteworthy.
[62]	1999–2008	Taiwan, China	Human	16.1% (10/76)	CIP, LVX, CAZ, CTX, FOX, CRO	GyrA mutations are the major quinolone resistance mechanisms in *Salmonella*. Overproduction of efflux pump and presence of *qnr* and *oqxAB* play additional roles.
[63]	2012–2013	Shenzhen, China	Retail meat (chicken, pork)	91% (75/82)	CTX, CIP, AZM, AMP, NAL, KAN, STR, CHL, TET	PMQRs greatly facilitate development of FQs resistance in *Salmonella*.
[61]	2010–2011	Fujian, Henan, Guangdong, Beijing, Guangxi, Shanxi, Sichuan, Shanghai China	Retail raw chicken carcasses	42% (194/462)	NAL, CIP, LVX, GAT	Isolates which harboured more PMQR genes and accumulated more point mutations on GyrA and ParC presented higher resistance levels to quinolones.
[15]	2007–2011	China	Poultry, swine, animal hospital	31.7% (20/63)	OLA, NAL, GEN, CHL, FFC, AMP, CIF, TET, SMX	All the isolates carring transferable IncHI2-type plasmids haboured *oqxAB* cassette and incomplete class 1 integron.
[64]	2015–2016	Shandong, Henan, Jiangsu, Anhui, Liaoning, Tianjin, Beijing, Hebei, Hubei, Guizhou, Xinjiang, Ningxia, Sichuan, China	Chicken	8.24% (14/170)	CTX, AK, CIP, AMP, CTI,FEP, ATM, STR, NAL, NOR, SXT, CHL, TET, DOX	The *bla*_CTX-M_ genes, 16S rRNA methylase genes (*armA*, *rmtD* or *rmtC*) and five plasmid-mediated quinolone resistance (PMQR) determinants (*aac(6′)-Ib-cr*, *oqxAB*, *qnrB*, *qepA* and *qnrD*) were identified in 18 *S.* Indiana and 17 *S.* California isolates.

Notes: *OLA* olaquindox, *CHL* chloramphenicol, *CIP* ciprofloxacin, *AMP* ampicillin, *KAN* kanamycin, *TET* tetracycline, *SXT* sulfamethoxazole-trimethoprim, *FFC* florfenicol, *LVX* levofloxacin, *CTX* cefotaxime, *CAZ* ceftazidime, *STR* streptomycin, *GEN* gentamicin, *CTI* ceftiofur, *DOX* doxycycline, *FOX* cefoxitin, *CRO* ceftriaxone, *CTX* cefotaxime, *AZM* azithromycin, *GAT* gatifloxacin, *AK* amikacin, *FEP* cefepime, *NAL* nalidixic acid

**Table 3:** Epidemiology of *oqxAB* in *Klebsiella pneumoniae*

References	Bacterial species	Year(s) of isolates collection	Geographic area	Sample source	Percentage of *oqxAB* carring isolates (no. of isolates)	Resistance phenotype(s)	Descriptions
[19]	*K. pneumoniae*	1998–2006	Seoul Korea	Human	*K. pneumoniae*: 74.1% (100/135)	OLA, CIP	This is the first report of the presence of an *oqxAB*-containing plasmid in a human isolate of *E. coli*.
[14]	*K. pneumoniae Klebsiella* spp*.*	2010	Shanghai China	Human	*K. pneumoniae*: 100% (154/154)	NAL, CIP, NOR, LVX, OLA, TMP, CHL, TET	Variants of *oqxA2*, *oqxB2* and *oqxB3* were identified.
[67]	*K. pneumoniae*	2005–2010	Seoul, Korea	Human	*K. pneumoniae*: 10.8% (11/102)	NAL, LVX, AK, GEN, TOB	The *oqxAB* gene was only found in *K. pneumoniae* isolates.
[71]	*K. pneumoniae,*	2010	Tunis	Human	*K. pneumoniae*: 65% (26/40)	GEN, TOB, AK, NAL, CIP, SXT	The prevalence of PMQR determinants among ESBL-producing strains
[8]	*K. pneumoniae*	2012	Northeast Ohio	Human	*K. pneumoniae*: 100% (36/36)	CIP	KPC-2 and KPC-3 types β-lactamase (bla) genes
[8]	*K. pneumoniae*	2006–2009	New York, New Jersey, Pennsylvania	Human	*K. pneumoniae*: 83.3% (5/6)	CIP	KPC-2 and KPC-3 types β-lactamase (bla) genes
[8]	*K. pneumoniae*	1996–1997	Taiwan, Turkey, Australia, South Africa, Argentina, Belgium, the US	Human	*K. pneumoniae*: 87.5% (14/16)	–	TEM-10, SHV-2, SHV-5, CTX-M-2, CTX-M-3 types β-lactamase (bla) genes
[68]	*K. pneumoniae*	2012–2014	Jiangsu, China	Human	*K. pneumoniae*: 67.6% (50/74)	AK, Q	Novel mutants (*oqxA11*, *oqxB13*, *oqxB27*, and *oqxB28*) were identified.
[69]	*K. pneumoniae*	2014–2015	Tehran, Iran	Human	*oqxA*: 56.7% (140/247), *oqxB*: 54.6% (135/247)	CAZ, NOR, CTX, CPD, NAL, CIP, GEN, TGC,AK	The predominant coexisting ESBL and PMQR profile among our isolates included *bla*_CTX-M_ and *aac(6′)-Ib*, *oqxA*, *oqxB* (28.3%) and *bla*_TEM_, *bla*_SHV_ and *aac(6′)-Ib*, *oqxA*, and *oqxB* (19.4%) profile.

Notes: *OLA* olaquindox, *CHL* chloramphenicol, *CIP* ciprofloxacin, *TET* tetracycline, *SXT* sulfamethoxazole-trimethoprim, *LVX* levofloxacin, *CTX* cefotaxime, *CAZ* ceftazidime, *GEN* gentamicin, *CTX* cefotaxime, *AK* amikacin, *TOB* tobramycin, *NAL* nalidixic acid, *CPD* cefpodoxime, *TGC* tigecycline, *Q* quinolones

Till now, several reviews have summarized the current knowledge of plasmid-mediated quinolone resistance (PMQR) genes, but there are no reviews specifically focused on the OqxAB efflux pump. A comprehensive understanding of the nature and epidemiology of this OqxAB efflux pump will benefit for future strategies of optimizing antimicrobial use and development of anti-resistance interventions. In this paper, a literature search was conducted using the online databases of Pubmed (www.ncbi.nlm.nih.gov/entrez/query.fcgi; 1809 until present) and ISI Web of Science (http://www.isiwebofknowledge.com; timespan 1945 until present) with no restriction on the date of publication. A total of 117 relevant publications were identified using the keyword “oqxAB”. Eighty-seven of the publications with close relevance to the nature and/or epidemiology of OqxAB efflux pump were included in this review as references. The updates on the genetic characteristics, prevalence of the OqxAB multidrug efflux pump in various bacterial species and its contribution on multidrug resistance were fully reviewed.

## Genetic characteristics and regulation of *oqxAB* operon

The plasmid-borne OqxAB efflux pump has been reported since 2004. Sørensen et al. isolated an *Escherichia coli* (*E. coli*) from swine manure in a farm using olaquindox as a feed additive [9]. Using agar dilution test with Sensititre plates, they found that this isolate was resistant or has reduced susceptibility to a number of antibiotics, including olaquindox (OLA; MIC 128 mg/L), carbadox (CBX; MIC 128 mg/L), ampicillin (AMP; MIC 32 mg/L), kanamycin (KAN; MIC 64 mg/L), chloramphenicol (CHL, MIC 64 mg/L), streptomycin (STR; MIC 128 mg/L), sulfamethoxazole (SXT; MIC 512 mg/L) and trimethoprim (TMP, MIC 32 mg/L). Through filter conjugation, the reduced susceptibility to ampicillin, chloramphenicol and olaquindox could be transferred to the recipient strain *E. coli* CSH26, while the resistance to kanamycin, streptomycin, sulfamethoxazole, trimethoprim and carbadox were not transferred. The transconjugants received a plasmid from the donor strain and this 52 kb plasmid was later named as pOLA52 [9].

**Fig. 1:**
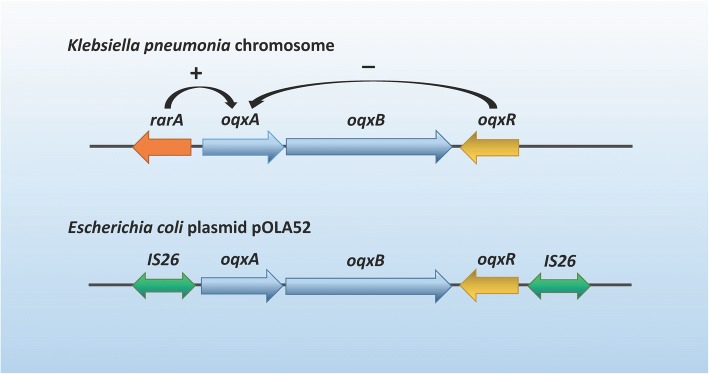
Genomic organization of the *rarA-oqxABR* locus on the chromosome of *Klebsiella pneumoniae* and *IS26- oqxABR* transposon on the plasmid of pOLA52 in *Escherichia coli*

**Fig. 2:**
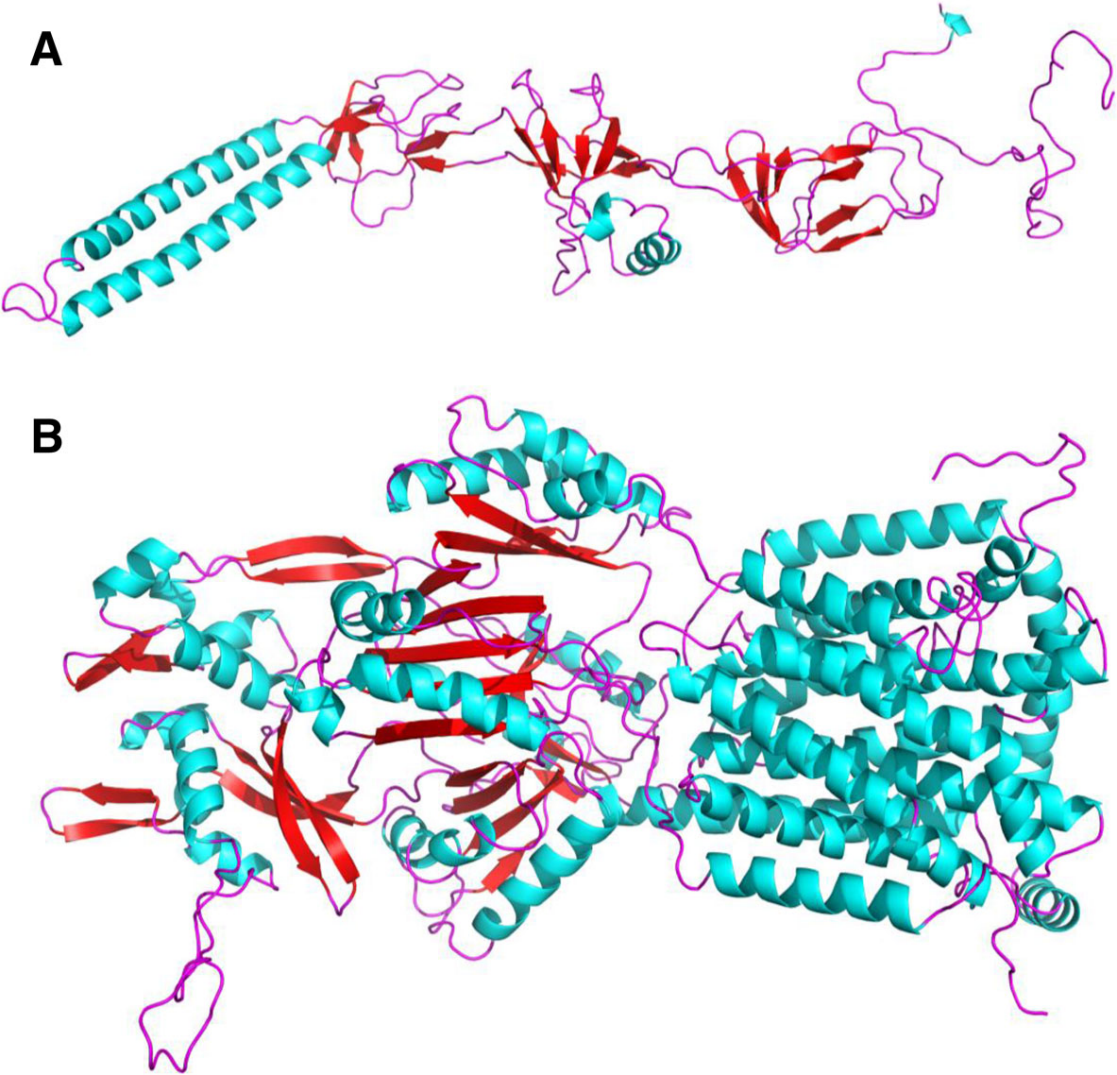
The structures of OqxA (**a**) and OqxB (**b**) protein predicted by homology modeling using SYBYL X-2.0

Subsequently, Hansen et al. [7] subcloned and sequenced the genetic elements of this conjugative plasmid pOLA52 and found the genetic elecments was composed of three open reading frames encoding putative proteins (Fig. 1). Two of the proteins designated as OqxA and OqxB were highly homologous to efflux pumps from the RND family in other bacterial species. By homology modeling using the software of SYBYL X-2.0, we found that the positions and numbers of the transmembrane helices in the crystal structure of OqxB was highly consistent with AcrB protein in *E. coli*, MexB protein in *Pseudomonas aeruginosa*, CmeB protein in *Campylobacter jejuni*, AdeB protein in *Acinetobacter baumannii* and MtrD protein in *Neisseria gonorrhoeae* (Fig. 2). The ORF3 was homologous to a putative transcriptional regulator and may be involved in the regulation of *oqxAB* operon. The plasmid pLOW2::*oqxAB* in *E. coli* strain N43 was found to be a H-driven ethidium bromide efflux and showed high-level resistance to olaquindox (OLA; MIC> 128 mg/L) and chloramphenicol (CHL; MIC> 64 mg/L), while the control plasmid (pLOW2) in *E.coli* N43 only presented low-level resistance to olaquindox (OLA; MIC 8 mg/L) [7]. This is the first report of a plasmid-encoded multidrug efflux pump conferring resistance to olaquindox [7].

A gene cassette (*mrkABCDF*) was also identified on the pOLA52, which was in relation to the bacterial functions of type 3 fimbriae expression and biofilm formation [10]. When the *mrk* operon was inactivated, the conjugative transfer ability of pOLA52 was also remarkably reduced. In 2008, the complete sequence of pOLA52 was analyzed and annotated [11]. The 51,602 bp conjugative plasmid contained 68 putative genes, which were associated with functions of conjugation, replication and partitioning. Half of these genes constituted a conserved IncX1-type backbone. A fragmented Tn*3* transposon encoded resistance to ampicillin. The RND family efflux pump encoding gene *oqxAB* and the biofilm formation involved gene *mrkABCDF* were located on two composite transposons (Tn*6010* and Tn*6011*) [11].

Tn*6010*-associated *oqxAB* was detected in *Klebsiella* spp. and *Raoultella* spp. by a reliable two-step PCR-based method [12]. Chromosome-borne *oqxAB* was identified in *Klebsiella* spp*.* and *Raoultella* spp*.* by hybridization with I-CeuI-restricted genomes [13]. A recent study of the evolution and dissemination routes of the *oqxAB*-like elements among different bacterial species found that the *oqxAB* gene was detected in the chromosome of all tested *K. pneumoniae*, even those isolated before the year of 1984, supporting that the plasmid-borne *oqxAB* was most likely to be acquired from the chromosome of *K. pneumoniae* and *Raoultella* spp*.* [13]. The sequence of chromosome-based *oqxAB* in *K. pneumoniae* was highly homologous to the sequence of plasmid-based *oqxAB* in *E. coli* (e.g pOLA52) and *Salmonella* isolates, while low homologous to that in *Enterobacter* spp. [13] Novel mutants named as *oqxA2*, *oqxB2* and *oqxB3* were identified in *K. pneumoniae* [14]. A number of plasmid replicons were able to transfer the *oqxAB* gene, including IncF, IncH, IncI, IncHI2 and IncX [11, 15–18]. IS26-like insertion sequences flanked the *oqxAB* operon and the local repressor gene *oqxR* might be mobilized as part of a 6731 bp composite transposon known as Tn*6010* [19] (Fig. 1). The *nimC* element, *blmS* bleomycin resistance gene and transposition elements IS26 and Tn*3* were located on the upstream of Tn*6010* in *Salmonella*, indicating the possibility of transposition of an entire 10-kb fragment from pOLA52 [20].

Many transcriptional regulators, like MarA, SoxS, RamA and Rob, have been reported to upregulate the expression of the RND type efflux pumps in *Enterobacteriaceae*, thus contributed to the MDR phenotype [21]. In *K. pneumoniae*, the regulatory mechanisms of OqxAB efflux pump have been extensively studied. In 2012, a novel chromosomally encoded AraC-type positive regulator (RarA, regulator of antibiotic resistance A) was identified from *K. pneumoniae*. Overexpression of *rarA* can upregulate expression levels of its downstream efflux pump operon *oqxAB* and *acrAB* [22]. The *rarA* gene was located on the genomes of several *Enterobacteriaceae*, such as *K. pneumoniae*, *Enterobacter* and *Serratia proteamaculans*. Plasmid-mediated overexpression of *rarA* can lead to MDR phenotype in either *E. coli* or *K. pneumoniae* without the presence of *rob*, *marA* or *soxS*, but requires the assistance of a functional AcrAB efflux pump. A transcriptome and phenotypic microarray study showed that *rarA* in *K. pneumoniae* may be associated with the functions of cell envelope biogenesis and posttranslational modification, transport proteins and the porin OmpF, and thus enhanced growth of the over expresser under the pressure of several antibiotic classes, i.e., minocycline, beta-lactams, polymyxin B, fluoroquinolones (FQs), furaltadone and sanguinarine [23]. Jiménez-Castellanos et al. confirmed that the OqxAB was regulated by RamA and RarA, while AcrAB efflux pump was regulated by RamA and SoxS, and the outer membrane protein TolC was controlled by all these regulators in *K. pneumoniae* [24]. RamA is the most effective transcriptional regulator of antibiotic susceptibility in *K. pneumoniae*, followed by RarA, SoxS, and MarA.

Another GntR-type regulator, *oqxR*, was generally located neighboring to the *oqxAB* and could decrease the expression of the OqxAB efflux pump. Several amino acid substitutions including Phe6Ser, Gln11Leu, Asp95Glu, Val113Ile, and frameshift deletion of amino acids 73 to 77 or positions 88 to 94, have been identified on the OqxR and may be detrimental to its function [22]. A novel amino acid substitution (Val102Gly) identified on the OqxR in a clinical isolate of *K. pneumoniae* could also induced the elevated expression of both *oqxAB* and *rarA* [25]. Complementation with wild-type *oqxR* can restore the susceptibility to antibiotics and normalized the *rarA* and *oqxAB* expression levels. Overactivation of the OqxAB efflux pump contributed to the MDR phenotype and enhanced virulence of this particular clinical isolate [25]. Taken together, *oqxAB* is subject to regulation by both RarA and RamA (AraC-type transcriptional activator) and OqxR (GntR-type transcriptional repressor).

The overexpression of *oqxAB* was found to confer more than 4-fold reduced susceptibility to a variety of antibiotics, including quinoxaline compounds (olaquindox and carbadox), chloramphenicol, quinolones and fluoroquinolones, and trimethoprim. Besides, the OqxAB multidrug efflux pump also contributed to the reduced susceptibility to detergents and disinfectants, including benzalkonium chloride, triclosan, especially SDS [26]. The *oqxAB* bearing plasmid pOLA52 could readily be transferred among *Enterobacteriaceae* and the transconjugants showed reduced susceptibility to chloramphenicol, ciprofloxacin and olaquindox [26]. The MDR phenotypes were attributed to the overexpression of the *oqxAB*, as illustrated by the gene expression analysis. Transposition of *oqxAB* gene from chromosome to plasmids was able to result in more than 80-fold increase of the OqxAB efflux pump expression level, thus leading to the MDR phenotypes [13]. Over the past decades, there have been increasing studies reporting the magnitudes of the OqxAB efflux pump’s contribution to reduced susceptibility to different classes of drugs, and the prevalence of *oqxAB* gene complex in various bacteria originated from human and animal sources (Tables 1, 2 and 3).

## Relationship of OqxAB efflux pump with quinoxaline resistance

OqxAB efflux pump has been the only known resistance genetic mechanism against quinoxalines till now. The quinoxaline representative drug olaquindox is generally used as a growth promoter on piglets at concentration up to 100 mg/kg per feed [9]. In European countries, the application of olaquindox was banned in 1999 due to its potential toxicity [27]. Furthermore, there have been serious concerns due to the possibility for selection of drug resistance since its introduction in the 1980s. An epidemiological study from Denmark in 1999 has demonstrated the presence of olaquindox resistant bacteria [9]. Other studies also reported that the increase of olaquindox resistance increased along with the usage of olaquindox as growth promoter in the pig farms [28, 29].

The *oqxA* gene was detected in olaquindox resistant *E. coli* collected from Danmark and Sweden from 1995 to 1998 [27]. In an epidemiological study from China, of all the 172 strains of *E. coli*, the *oqxAB* gene was detected in 46.3% of the isolates from swine, 13% from chicken, 43.9% from farm environment and 30.3% from farmworkers [17]. In comparision to the *oqxAB*-negative isolates, the MIC_50_ values of quinoxalines (mequindox and olaquindox) were 8- to 32-fold higher in the *oqxAB*-positive isolates [17]. In a recent study, the *oqxAB* prevalence among animals isolates (33.39%) was much higher than food (17.34%) and human (18.07%) isolates [30]. Another study from Henan province in China showed that *oqxAB* were observed in 58.5% (62/106) of the *E. coli* isolates from dogs, 56.25% (36/64) from cats, and 42.0% (42/100) from human patients [31]. The MIC_50_ values of olaquindox against *oqxAB*-positive isolates were 4- to 16-fold higher than those of the *oqxAB*-negative isolates [31]. These studies demonstrated that the OqxAB multidrug efflux pump significantly contributed to the reduced susceptibility or resistance to olaquindox.

The *oqxAB* was also reported to be responsible for the reduced susceptibility to another quinoxaline drug mequindox in *E. coli*. Of all the 1123 strains of enteropathogenic *Escherichia coli* (EPEC) isolated from farm animals in China from 1970s to 2013, *oqxAB* gene cassete was detected in 94.4% (322/341) of the high-level resistant strains (MIC_MEQ_ ≥ 64 mg/L) and 1.2% (9/782) of the low-level resistant isolates with mequindox MICs≤32 mg/L [32]. In addition, the circular intermediate of IS26-*oqxAB* was deteced in 93.4% of the *oqxAB*-positive strains, suggesting that the *oqxAB*-carring transposon Tn*6010* was unstable and tend to excision through the IS26-mediated recombination [32].

## Contribution of OqxAB efflux pump to quinolone resistance

Most of the studies regarding *oqxAB* have focused on the contribution of OqxAB efflux pump to the quinolone resistance, since quinolone and FQs antibiotics are one of the most critically important classes of broad spectrum anti-infective agents which are used to treat various bacterial infections in both humans and animals [33]. The mechanisms of quinolone resistance mainly consists of target-site gene mutations that lead to amino acid substitutions in the quinolone resistance-determining regions (QRDRs) of the topoisomerase unit, reduced intracellular drug accumulation by overexpression of multidrug efflux pumps or decreased permeability of outer membrane porins, and plasmid-mediated quinolone resistance mechanisms [34].

The *oqxAB* has been recognized as one of the four genetic mechanisms of the PMQRs in recent years and there is a lot of data with regard to its contribution to the FQ resistance phenotype (Tables 1, 2 and 3). As demonstrated by RT-PCR, the expression levels of *oqxAB* in ESBL-producing *K. pneumoniae* with reduced susceptibility to quinolones were 4-fold higher than the susceptible strains [35]. Knocking out the *oqxAB*-encoding plasmid using homologous recombination in a FQ-resistant clinical *E. coli* strain with no mutations on the QRDRs of DNA gyrase and topoisomerase IV could decrease FQ MICs by 2- or 4-fold, and the OqxAB efflux pump may also work in conjuction with TolC to mediating the decreased FQ resistance [36]. Wong et al. cloned *oqxAB* gene into a plasmid (pTrc) and transformed into *Salmonella typhimurium* strain LT2, and found that acquisition of the *oqxAB*-encoding plasmid by other two *S. typhimurium* isolates (strain 11–28 and strain 10–63) caused a 4-fold increase of MIC to ciprofloxacin and also conferred resistance to streptomycin, ampicillin, tetracycline, chloramphenicol, trimethoprim, sulfamethoxazole, nalidixic acid and olaquindox [37].

The OqxAB efflux pump could not only mediate low-level quinolone resistance, but also is important for the bacteria to survive under low concentration of FQs and facilitate the subsequent topoisomerase mutations associated with higher level resistance [38]. In comparison to the Qnr-producing isolates, the frequencies of topoisomerase mutations in *oqxAB*- and *qepA*-carring strains were relatively higher [39]. A recent study showed that acquisition of *oqxAB*-carring plasmids by *E. coli* led to a 4–8 fold increase in the MIC and a 8–16 fold elevation of the mutation prevention concentration (MPC) to ciprofloxacin. Meanwhile, the development of FQ resistance was faster in the transformants bearing the *oqxAB*-carring plasmids than that in the parental strain, particularly before 16 passages [40]. The *oqxAB*-carring plasmid in *E.coli* induced a fitness cost in vitro, however, the biological benefits were observed in vivo and their fitness was increased when combined with chromosomal mutations [40]. However, the growth of *oqxAB*-bearing *Salmonella* was much weaker than the parent strain, indicating the fitness cost of acquisition of *oqxAB* in *Salmonella* [37].

The *oqxAB* gene has been frequently detected in quinolone resistant bacteria, such as *E. coli* (Table 1), *Salmonella* (Table 2) and *K. pneumoniae* (Table 3). The prevalence of *oqxAB* in *E. coli* of human origin was firstly reported in 2009 and relatively lower than that of the animal or environmental sources [19]. The *oqxAB* genes were 1 (0.4%) of 261 strains of *E. coli*, 3 (4.6%) of 65 strains of *Enterobacter cloacae*, and 100 (74.1%) of 135 strains of *K. pneumoniae* recovered from blood samples collected from patients in Seoul National University Hospital from 1998 to 2006 [19]. Two novel mutants of *oqxB* (*oqxB20* and *oqxB29*) were also identified *E.coli* in China [41]. The first detection of *oqxAB*-carring *E. coli* in Latin America was in 2017 from Bolivia [42]. The *oqxAB* (*n* = 15) and *aac(6′)-Ib-cr* (*n* = 14) were the most prevalent PMQR genes in 248 strains of levofloxacin resistant *E. coli* collected from a university hospital in Taiwan from 2001 to 2015 [43]. Of 190 strains of *E. coli* collected from a rural and a urban hospital wastewaters in India, two isolates were found to be *oqxAB*-positive, emphasizing the importance to dispose the wastewater properly to reduce the pollution of environment with resistance genes and resistant bacteria [44].

In comparison to the low prevalence of *oqxAB* in *E. coli* of human origin, the frequency of *oqxAB* in animal isolates is relatively high. The *oqxAB* was even detected in *E. coli* isolated from ducks, geese and as early as 1994 from chickens [45]. The presence of *oqxAB* in *E. coli* from farm and wild lagomorphs was reported in Italy in the 113 strains of multi-drug resistant *E. coli* collected from 2006 to 2008 [46]. The dissemination of *oqxAB* located on a transferable plasmid pHXY0908 between *Salmonella* and *E. coli* in the chickens gut was observed under the treatment of chickens with enrofloxcin [47]. Several investigation data showed that in China the prevalence of *oqxAB* was in 19–51% in swine and chicken, which was higher than the prevalence of other PMQR genes (*qnr* 5.7–13.6%, *aac(6′)-Ib-cr* 4.9–11.6% and *qepA* 2.6–3.64%) in animal original strains, and much higher the prevalence of *oqxAB* in human strains [45, 48–50].

Many reports emphasized the co-spread of *oqxAB* with other resistance genes, virulence genes and heavy metal resistance genes in *E.coli*. The sequences of several plasmids from foodborne *E. coli*, such as pEC012 [51], pE80 [52] and p42–2 [53] had been published in the Genebank. These conjugative plasmids harbored multiple resistance determinants, including *oqxAB*, *bla*_CTX-M-65_, *rmtB*, *fosA3* and *floR*, indicating a therapeutic challenge due to co-selection by various antibiotics and thus compromise the effectiveness of current antimicrobial strategies. In 2011, Liu et al. described co-existence of PMQR genes (*oqxAB* and *aac(6′)-Ib-cr*), ESBLs encoding gene (*bla*_CTX-M-24_) and a virulence determinant *iroN* on the same plasmid in an *E. coli* strain a6 [54]. They further reported the co-dissemination of *oqxAB*, *bla*_CTX-M_ and *floR* on the similar plasmids (F33:A-: B- and HI2) [16] and *oqxAB, bla*_DHA-1_, and 16S rRNA methylase gene *rmtB* on the same plasmid JGDA2 [55]. In *E. coli* isolated from pigeon farms in China in 2011–2012, the PMQRs (*oqxAB* and *aac(6′)-Ib-cr*), CTX-M type ESBLs encoding genes (CTX-M-65, CTX-M-27 and CTX-M-55) and *rmtB* were found to be co-transferred by IncF plasmid alone or in combination with the IncK [56]. The co-existence of *oqxAB*, *bla*_CTX-M_ and other antibiotic resistance genes with the *pco* and *sil* operons, which contributed to the increase of the MICs to CuSO_4_ and AgNO_3_ on IncHI2 plasmids, were also observed [57]. The co-existence of antibiotic resistance genes and heavy metal resistance genes might promote the development of multidrug resistant bacteria when exposed to heavy metals, particularly copper and zinc, which were commonly used as growth promoters for some livestock species.

The first report of *oqxAB* presence in *Salmonella* was in 2012 and the *oqxAB* gene associated with IS26 was located on the chromosome of two strains of *Salmonella* Derby isolated from pork samples in Hong Kong [58]. The *oqxAB* gene was detected in 112 (72.73%) of 154 strains of *S. enterica* serovar Indiana recovered from animals (*n* = 133) and humans (*n* = 21) in China from 2009 to 2013 and showed concurrent resistance to both ciprofloxacin and cefotaxime [59]. The 12 (2%) of 546 strains of human clinical *Salmonella* Typhimurium collected from patients in China from 2005 to 2011 were co-resistant to both ciprofloxacin and ceftriaxone, four of the 12 resistant isolates carried *oqxAB* gene and a single *gyrA* (D87N) mutation [60]. The *oqxAB* (42.0%) was found in 462 strains of FQ-resistant *Salmonella* collected from retail chicken meat in China during 2010–2011 [61]. The prevalence of *oqxAB* was 16.1% in the 76 strains of cephalosporins resistant and quinolones resistant *Salmonella* identified from 1416 clinical isolates in Taiwan from 1999 to 2008 [62]. Among 82 *Salmonella* collected from food in Shenzhen of China from October 2012 to June 2013, which showed co-resistance to three critically important antibiotics (ceftriaxone, 10%; ciprofloxacin, 39%; azithromycin, 25%), the *oqxAB* (91%), *aac(6′)-Ib-cr* (75%) and *qnrS* (66%) were frequently detected [63]. The combination of *oqxAB* and a single target mutation on GyrA could possibly mediate development of resistance to ciprofloxacin in *Salmonella*, and dramatically reduced the time required for the development of a resistance associated with generation of double GyrA mutations and single ParC mutation [63].

In *Salmoenlla*, the *oqxAB* was also found to be co-spread with other resistance genes. In 2013, Li et al. reported that the *aac(6′)-Ib-cr* (36.5%), *oqxAB* (31.7%) and *qnrS1* (1.6%) were present alone or in combination in 63 strains of *S.* Typhimurium isolates collected from animals in China and the *oqxAB* and *aac(6′)-Ib-cr* genes were found to be located on the same IncHI2-type plasmids [15]. Of 170 strains of *Salmonella* isolates recovered from 1540 chicken samples in China from 2015 to 2016, the resistance rates of ciprofloxacin, amikacin and cefotaxime were as high as 68.2, 48.2 and 44.7%, respectively and *oqxAB* (8.24%) and *aac(6′)-Ib-cr* (15.88%) were the most prevalent PMQR genes [64]. Recently, the co-spread of *oqxAB* and *mcr-1*, which conferred resistance to colistin, were also found on a transferable IncI2 plasmid in *S.* Typhimurium /ST34 [65].

The wide distribution of *oqxAB* was found among the clinical isolates of MDR *K. pneumoniae*, which is a frequent nosocomial pathogen and causes life-threatening disease in humans [66]. In an international report of ESBL-producing *K. pneumoniae*, the *oqxAB* gene was detected in 87.5% (14/16) of the isolates collected from Hospitals in Taiwan, Australia, Argentina, Belgium, Turkey, South Africa, and the United States from 1996 to 1997 [8]. For *K. pneumoniae* isolates collected from the mid-Atlantic, *oqxAB* was observed in 71% (22/31) of the ST 258 isolates and 91.7% (11/12) of the non-ST 258 isolates, respectively. The *oqxAB* was 100% detected among both quinolone resistant and quinolone-susceptible KPC-producing *K. pneumoniae* recovered from northeast Ohio [8]. An epidemiology study from China reported that 66.9% of the *K. pneumoniae* were resistant to ciprofloxacin and the *oqxAB* gene was detected in all the *K. pneumoniae* isolates [14]. Of 22 nonduplicate strains of ciprofloxacin-nonsusceptible *K. pneumoniae* (*n* = 22) recovered from blood samples of patients at a tertiary hospital in Korea from 2005 to 2010, *oqxAB* was detected in 11 (50%) strains of *K. pneumoniae* [67]. Of 74 carbapenemase-producing *K. pneumoniae* collected from a tertiary hospital in China from 2012 to 2014, *oqxAB* was detected in 48 FQ-resistant and 2 FQ-susceptible *K. pneumoniae* isolates and Variants of *oqxA11*, *oqxB13*, *oqxB27* and *oqxB28* were identified [68]. In the 247 strains of ESBLs-producing *K. pneumoniae* from 750 patients with nosocomial urinary tract infection (UTI), *oqxA* and *oqxB* were 56.7 and 54.6% of these isolates [69]. Among the 40 strains of ciprofloxacin resistant *E. coli* and 50 strains of ciprofloxacin resistant ESBLs-producing *Klebsiella* spp. isolated from blood samples in Hungary, the *oqxA* and *oqxB* were 48 and 40% of the isolates, respectively [70]. Among the 32 strains of *K. pneumoniae* from Tunisian hospital, *oqxAB* was observed in 65% of the *K. pneumoniae* [71]. *OqxAB* was detected on the chromosome of a hypermucoviscous, multidrug resistant, biofilm producing *K. pneumoniae* strain U25 from India, as demonstrated by whole genome sequencing [72]. *OqxAB* was also detected on the plasmid of IncR with other resistance genes like *bla*_NDM-1_ in two carbapenem resistant *K. pneumoniae* isolates collected from urine samples in a patient hospitalized at Annaba University hospital (Algeria) in 2014 [73].

## Role of OqxAB efflux pump in tigecycline resistance

Tigecycline is one of the few effective therapeutic options for treating serious infections caused by MDR pathogens, such as *K. pneumoniae* [74]. Resistance mechanisms of tigecycline have been comprehensively studied. Overexpression of multidrug efflux pumps such as AcrAB in *E. coli* [75], *K. pneumoniae* [74], and *Enterobacter cloacae* [76]; AdeABC in *Acinetobacter baumanni* [77], and MexXY in *Pseudomonas aeruginosa* [78] have been implicated to contribute to tigecycline resistance. For *K. pneumoniae* isolates with MICs no more than 8 mg/ml, AcrAB-TolC efflux pump played the most important role in the tigecycline resistant *K. pneumoniae*, while for strains with MICs≥16 mg/ml, both AcrAB-TolC and OqxAB efflux pumps are required [74]. OqxAB efflux pump was also associated with the heteroresistance to tigecycline in *Salmonella*, which was attributed to the overexpression of AcrAB-TolC and OqxAB efflux pumps, since PAβb restored the susceptibility to tigecycline in heteroresistant isolates and accumulation of tigecycline in cell was also reduced [79]. Veleba and Schneiders verified the important roles of *ramA* and *rarA* on the overexpression of efflux pump encoding genes *acrAB* and *oqxAB*, and its contribution to the tigecycline resistance in *K. pneumoniae*, *Enterobacter cloacae* and *Enterobacter* aerogenes [22, 80]. Higher transcription levels of *rarA* in conjunction with *oqxB* were also observed in tigecycline resistant *K. pneumoniae* isolates in comparison with a tigecycline-susceptible strain [74]. By studying five isogenic paired clinical isolates of *K. pneumoniae* identified from same patient in a medical center in Taiwan, the researchers found that acquisition of tigecycline resistance in *K. pneumoniae* did not necessarily compromise their virulence and growth [81]. They further reported that bacteremia, caused by infection with tigecycline-nonsusceptible *K. pneumoniae* was linked to high mortality, and previous medication of fluoroquinolones was the independent risk factor for the selection of tigecycline resistance in *K. pneumoniae* [82].

## Role of OqxAB efflux pump in nitrofurantoin resistance

Nitrofurantoin is a valuable antibiotic used to treat acute uncomplicated urinary tract infections [83]. Resistance to nitrofurantoin in *E. coli* was primarily due to mutations in the nitroreductase genes (*nfsA* and *nfsB*), which participated in the converting of antibiotics into toxic intermediate compounds [84]. Recently, plasmid-mediated *oqxAB* has been reported to be an important mechanism for nitrofurantoin (NIT) resistance [85]. Ho et al. investigated the molecular epidemiology of *oqxA* and *oqxB* and its relationships with nitrofurantoin resistance in 341 strains of *E. coli*, which were recovered from patients suffered from UTI (*n* = 205; collected in 2004 to 2013) and animals (*n* = 136; collected in 2012 to 2013) [85]. They found that the prevalence of *oqxAB* gene was significantly higher in the NIT-intermediate (11.5–45.5%) and NIT-resistant (39.2–65.5%) isolates than that in the NIT-susceptible strain (0–1.7%). In the 20 NIT-intermediate/resistant *E. coli* isolates, curing of the *oqxAB*-bearing plasmids could lead to the reduction of geometric mean MIC_(NIT)_ from 168.9 g/ml to 34.3 g/ml. Acquisition of the plasmid-borne *oqxAB* could result in a 2 to 16 fold elevation of the MIC_(NIT)_ and increase the mutation prevention concentration of NIT from 128 g/ml to 256 g/ml. The combination of *oqxAB* and *nfsA* (chromosomal nitroreductase gene) mutations was sufficient to confer clinically important high-level NIT resistance in *E. coli* [85].

## Conclusions

Since the discovery of *oqxAB* in 2003, the gene has been reported to locate on different plasmids with other antimicrobial resistance genes and virulence determinants in various bacteria, especially in *E. coli*, *Salmonella* and *Enterobacter aerogenes*. The plasmid-borne *oqxAB* was most likely to be originated from the chromosome of *K. pneumoniae*. The OqxAB efflux pump significantly contributed to reduced susceptibility to olaquindox, tigecycline, nitrofurantoin and chloramphenicol, and facilitated the development of high-level fluoroquinolone resistance. There is a great need to monitor the potential dissemination of the *oqxAB* gene among humans, animals and environment. Further investigation and understanding of the natural functions, determinants of mobilization, and the regulation of expression of the OqxAB efflux pump will aid in future strategies of antimicrobial usage optimization.

## Abbreviations

AMP: Ampicillin
CBX: Carbadox
CHL: Chloramphenicol
*E. coli*: *Escherichia coli*
EPEC: Enteropathogenic *Escherichia coli*
FQs: fluoroquinolones
*K. pneumoniae*: *Klebsiella pneumoniae*
KAN: Kanamycin
MDR: Multidrug resistance
MIC: Minimal inhibitory concentration
MIC_50_: Minimum inhibitory concentration of the antibiotic for inhibiting 50% of the isolates
MPC: Mutation prevention concentration
NIT: Nitrofurantoin
OLA: Olaquindox
PMQR: Plasmid-mediated quinolone resistance
QRDRs: Quinolone resistance-determining regions
RND: Resistance nodulation and cell division
RT-PCR: Real time polymerase chain reaction
STR: Streptomycin
SXT: Sulfamethoxazole
TMP: Trimethoprim
UTI: Urinary tract infection

## References

[1] Holmes AH, Moore LSP, Sundsfjord A, Steinbakk M, Regmi S, Karkey A, Guerin PJ, Piddock LJV (2016). Understanding the mechanisms and drivers of antimicrobial resistance. Lancet.

[2] Sun J, Deng Z, Yan A (2014). Bacterial multidrug efflux pumps: mechanisms, physiology and pharmacological exploitations. Biochem Biophys Res Commun.

[3] Hernando-Amado S, Blanco P, Alcalde-Rico M, Corona F, Reales-Calderon JA, Sanchez MB, Martinez JL (2016). Multidrug efflux pumps as main players in intrinsic and acquired resistance to antimicrobials. Drug Resist Updates.

[4] Blair JMA, Richmond GE, Piddock LJV (2014). Multidrug efflux pumps in gram-negative bacteria and their role in antibiotic resistance. Future Microbiol.

[5] Nakaminami H, Noguchi N, Sasatsu M (2010). Fluoroquinolone efflux by the plasmid-mediated multidrug efflux pump QacB variant QacBIII in *Staphylococcus aureus*. Antimicrob Agents Chemother.

[6] Tauch A, Schluter A, Bischoff N, Goesmann A, Meyer F, Puhler A (2003). The 79,370-bp conjugative plasmid pB4 consists of an IncP-1beta backbone loaded with a chromate resistance transposon, the *strA*-*strB* streptomycin resistance gene pair, the oxacillinase gene Bla (NPS-1), and a tripartite antibiotic efflux system of the resistance-nodulation-division family. Mol Gen Genomics.

[7] Hansen LH, Johannesen E, Burmolle M, Sorensen AH, Sorensen SJ (2004). Plasmid-encoded multidrug efflux pump conferring resistance to olaquindox in *Escherichia coli*. Antimicrob Agents Chemother.

[8] Perez F, Rudin SD, Marshall SH, Coakley P, Chen L, Kreiswirth BN, Rather PN, Hujer AM, Toltzis P, van Duin D, Paterson DL, Bonomo RA (2013). OqxAB, a quinolone and olaquindox efflux pump, is widely distributed among multidrug-resistant *Klebsiella pneumoniae* isolates of human origin. Antimicrob Agents Chemother.

[9] Sorensen AH, Hansen LH, Johannesen E, Sorensen SJ (2003). Conjugative plasmid conferring resistance to olaquindox. Antimicrob Agents Chemother.

[10] Burmolle M, Bahl MI, Jensen LB, Sorensen SJ, Hansen LH (2008). Type 3 fimbriae, encoded by the conjugative plasmid pOLA52, enhance biofilm formation and transfer frequencies in *Enterobacteriaceae* strains. Microbiology.

[11] Norman A, Hansen LH, She Q, Sorensen SJ (2008). Nucleotide sequence of pOLA52: a conjugative IncX1 plasmid from *Escherichia coli* which enables biofilm formation and multidrug efflux. Plasmid.

[12] Guillard T, Lebreil AL, Hansen LH, Kisserli A, Berger S, Lozniewski A, Alauzet C, de Champs C (2015). Discrimination between native and Tn6010-associated *oqxAB* in *Klebsiella* spp., *Raoultella* spp., and other *Enterobacteriaceae* by using a two-step strategy. Antimicrob Agents Chemother.

[13] Wong MHY, Chan EWC, Chen S (2015). Evolution and dissemination of OqxAB-like efflux pumps, an emerging quinolone resistance determinant among members of *Enterobacteriaceae*. Antimicrob Agents Chemother.

[14] Yuan JY, Xu XG, Guo QL, Zhao X, Ye XY, Guo Y, Wang MG (2012). Prevalence of the *oqxAB* gene complex in *Klebsiella pneumoniae* and *Escherichia coli* clinical isolates. J Antimicrob Chemother.

[15] Li L, Liao X, Yang Y, Sun J, Li L, Liu B, Yang S, Ma J, Li X, Zhang Q, Liu Y (2013). Spread of *oqxAB* in *Salmonella enterica* serotype typhimurium predominantly by IncHI2 plasmids. J Antimicrob Chemother.

[16] Liu BT, Yang QE, Li L, Sun J, Liao XP, Fang LX, Yang SS, Deng H, Liu YH (2013). Dissemination and characterization of plasmids carrying *oqxAB*-*bla* CTX-M genes in *Escherichia coli* isolates from food-producing animals. PLoS One.

[17] Zhao J, Chen Z, Chen S, Deng Y, Liu Y, Tian W, Huang X, Wu C, Sun Y, Sun Y, Zeng Z, Liu JH (2010). Prevalence and dissemination of *oqxAB* in *Escherichia coli* isolates from animals, farmworkers, and the environment. Antimicrob Agents Chemother.

[18] Li L, Liao XP, Liu ZZ, Huang T, Li X, Sun J, Liu BT, Zhang Q, Liu YH (2014). Co-spread of *oqxAB* and *bla*CTX-M-9G in non-Typhi *Salmonella enterica* isolates mediated by ST2-IncHI2 plasmids. Int J Antimicrob Agents.

[19] Kim HB, Wang M, Park CH, Kim EC, Jacoby GA, Hooper DC (2009). *oqxAB* encoding a multidrug efflux pump in human clinical isolates of *Enterobacteriaceae*. Antimicrob Agents Chemother.

[20] Wong MH, Chan EW, Xie L, Li R, Chen S (2016). IncHI2 plasmids are the key vectors responsible for *oqxAB* transmission among *Salmonella* species. Antimicrob Agents Chemother.

[21] Duval V, Lister IM (2013). MarA, SoxS and rob of *Escherichia coli* - global regulators of multidrug resistance, virulence and stress response. Int J Biotechnol Wellness Ind.

[22] Veleba M, Higgins PG, Gonzalez G, Seifert H, Schneiders T (2012). Characterization of RarA, a novel AraC family multidrug resistance regulator in *Klebsiella pneumoniae*. Antimicrob Agents Chemother.

[23] De Majumdar S, Veleba M, Finn S, Fanning S, Schneiders T (2013). Elucidating the regulon of multidrug resistance regulator RarA in *Klebsiella pneumoniae*. Antimicrob Agents Chemother.

[24] Jimenez-Castellanos JC, Wan Ahmad Kamil WN, Cheung CH, Tobin MS, Brown J, Isaac SG, Heesom KJ, Schneiders T, Avison MB (2016). Comparative effects of overproducing the AraC-type transcriptional regulators MarA, SoxS, RarA and RamA on antimicrobial drug susceptibility in *Klebsiella pneumoniae*. J Antimicrob Chemother.

[25] Bialek-Davenet S, Lavigne JP, Guyot K, Mayer N, Tournebize R, Brisse S, Leflon-Guibout V, Nicolas-Chanoine MH (2015). Differential contribution of AcrAB and OqxAB efflux pumps to multidrug resistance and virulence in *Klebsiella pneumoniae*. J Antimicrob Chemother.

[26] Hansen LH, Jensen LB, Sorensen HI, Sorensen SJ (2007). Substrate specificity of the OqxAB multidrug resistance pump in *Escherichia coli* and selected enteric bacteria. J Antimicrob Chemother.

[27] Hansen LH, Sorensen SJ, Jorgensen HS, Jensen LB (2005). The prevalence of the OqxAB multidrug efflux pump amongst olaquindox-resistant *Escherichia coli* in pigs. Microb Drug Resist.

[28] Hedges AJ, Linton AH (1988). Olaquindox resistance in the coliform flora of pigs and their environment: an ecological study. J Appl Bacteriol.

[29] Linton AH, Hedges AJ, Bennett PM (1988). Monitoring for the development of antimicrobial resistance during the use of olaquindox as a feed additive on commercial pig farms. J Appl Bacteriol.

[30] Wang J, Guo Z-W, Zhi C-P, Yang T, Zhao J-J, Chen X-J, Zeng L, Lv L-C, Zeng Z-L, Liu J-H (2017). Impact of plasmid-borne *oqxAB* on the development of fluoroquinolone resistance and bacterial fitness in *Escherichia coli*. J Antimicrob Chemother.

[31] Liu B, Wu H, Zhai Y, He Z, Sun H, Cai T, He D, Liu J, Wang S, Pan Y, Yuan L, Hu G (2018). Prevalence and molecular characterization of *oqxAB* in clinical *Escherichia coli* isolates from companion animals and humans in Henan Province, China. Antimicrob Resist Infect Control.

[32] He T, Wang Y, Qian M, Wu C (2015). Mequindox resistance and *in vitro* efficacy in animal-derived *Escherichia coli* strains. Vet Microbiol.

[33] Aldred KJ, Kerns RJ, Osheroff N (2014). Mechanism of quinolone action and resistance. Biochemistry.

[34] Correia S, Poeta P, Hebraud M, Capelo JL, Igrejas G (2017). Mechanisms of quinolone action and resistance: where do we stand?. J Med Microbiol.

[35] Rodriguez-Martinez JM, Diaz de Alba P, Briales A, Machuca J, Lossa M, Fernandez-Cuenca F, Rodriguez Bano J, Martinez-Martinez L, Pascual A (2013). Contribution of OqxAB efflux pumps to quinolone resistance in extended-spectrum-beta-lactamase-producing *Klebsiella pneumoniae*. J Antimicrob Chemother.

[36] Sato T, Yokota S-I, Uchida I, Okubo T, Usui M, Kusumoto M, Akiba M, Fujii N, Tamura Y (2013). Fluoroquinolone resistance mechanisms in an *Escherichia coli* isolate, HUE1, without quinolone resistance-determining region mutations. Front Microbiol.

[37] Wong MH, Chan EW, Liu LZ, Chen S (2014). PMQR genes *oqxAB* and *aac(6')Ib-cr* accelerate the development of fluoroquinolone resistance in *Salmonella typhimurium*. Front Microbiol.

[38] Piddock LJV (2006). Clinically relevant chromosomally encoded multidrug resistance efflux pumps in bacteria. Clin Microbiol Rev.

[39] Tran JH, Jacoby GA, Hooper DC (2005). Interaction of the plasmid-encoded quinolone resistance protein Qnr with *Escherichia coli* DNA gyrase. Antimicrob Agents Chemother.

[40] Wang J, Zhi CP, Chen XJ, Guo ZW, Liu WL, Luo J, Huang XY, Zeng L, Huang JW, Xia YB, Yi MY, Huang T, Zeng ZL, Liu JH. Characterization of *oqxAB* in *Escherichia coli* isolates from animals, retail meat, and human patients in Guangzhou, China. Front Microbiol. 2017;8. 10.3389/fmicb.2017.01982 PMC5645526 29081769

[41] Zhao L, Zhang J, Zheng B, Wei Z, Shen P, Li S, Li L, Xiao Y (2015). Molecular epidemiology and genetic diversity of fluoroquinolone-resistant *Escherichia coli* isolates from patients with community-onset infections in 30 Chinese county hospitals. J Clin Microbiol.

[42] Saba Villarroel PM, Gutkind GO, Di Conza JA, Radice MA (2017). First survey on antibiotic resistance markers in *Enterobacteriaceae* in Cochabamba, Bolivia. Rev Argent Microbiol.

[43] Kao C-Y, Wu H-M, Lin W-H, Tseng C-C, Yan J-J, Wang M-C, Teng C-H, Wu J-J (2016). Plasmid-mediated quinolone resistance determinants in quinolone-resistant *Escherichia coli* isolated from patients with bacteremia in a university hospital in Taiwan, 2001-2015. Sci Rep.

[44] Chandran SP, Diwan V, Tamhankar AJ, Joseph BV, Rosales-Klintz S, Mundayoor S, Lundborg CS, Macaden R (2014). Detection of carbapenem resistance genes and cephalosporin, and quinolone resistance genes along with *oqxAB* gene in *Escherichia coli* in hospital wastewater: a matter of concern. J Appl Microbiol.

[45] Chen X, Zhang W, Pan W, Yin J, Pan Z, Gao S, Jiao X (2012). Prevalence of *qnr*, *aac(6′)-Ib-cr*, *qepA*, and *oqxAB* in *Escherichia coli* isolates from humans, animals, and the environment. Antimicrob Agents Chemother.

[46] Dotto G, Giacomelli M, Grilli G, Ferrazzi V, Carattoli A, Fortini D, Piccirillo A (2014). High prevalence of *oqxAB* in *Escherichia coli* isolates from domestic and wild lagomorphs in Italy. Microb Drug Resist (Larchmont, N Y).

[47] Chen Y, Sun J, Liao X-P, Shao Y, Li L, Fang L-X, Liu Y-H (2016). Impact of enrofloxacin and florfenicol therapy on the spread of OqxAB gene and intestinal microbiota in chickens. Vet Microbiol.

[48] Yang T, Zeng Z, Rao L, Chen X, He D, Lv L, Wang J, Zeng L, Feng M, Liu J-H (2014). The association between occurrence of plasmid-mediated quinolone resistance and ciprofloxacin resistance in *Escherichia coli* isolates of different origins. Vet Microbiol.

[49] Liu BT, Liao XP, Yang SS, Wang XM, Li LL, Sun J, Yang YR, Fang LX, Li L, Zhao DH, Liu YH (2012). Detection of mutations in the *gyrA* and *parC* genes in *Escherichia coli* isolates carrying plasmid-mediated quinolone resistance genes from diseased food-producing animals. J Med Microbiol.

[50] Xu X, Cui S, Zhang F, Luo Y, Gu Y, Yang B, Li F, Chen Q, Zhou G, Wang Y, Pang L, Lin L (2014). Prevalence and characterization of cefotaxime and ciprofloxacin co-resistant *Escherichia coli* isolates in retail chicken carcasses and ground pork, China. Microb Drug Resist (Larchmont, N Y).

[51] Pan Y-S, Zong Z-Y, Yuan L, Du X-D, Huang H, Zhong X-H, Hu G-Z (2016). Complete sequence of pEC012, a multidrug-resistant IncI1 ST71 plasmid carrying *bla* CTX-M-65, *rmtB*, *fosA3*, *floR*, and *oqxAB* in an avian *Escherichia coli* ST117 strain. Front Microbiol.

[52] Wong MH, Xie M, Xie L, Lin D, Li R, Zhou Y, Chan EW, Chen S (2016). Complete sequence of a F33:A-:B- conjugative plasmid carrying the *oqxAB*, *fosA3*, and *bla*CTX-M-55 elements from a foodborne *Escherichia coli* strain. Front Microbiol.

[53] Yang QE, Walsh TR, Liu BT, Zou MT, Deng H, Fang LX, Liao XP, Sun J, Liu YH (2016). Complete sequence of the FII Plasmid p42–2, carrying *bla*CTX-M-55, *oqxAB*, *fosA3*, and *floR* from *Escherichia coli*. Antimicrob Agents Chemother.

[54] Liu B-T, Wang X-M, Liao X-P, Sun J, Zhu H-Q, Chen X-Y, Liu Y-H (2011). Plasmid-mediated quinolone resistance determinants *oqxAB* and *aac(6′)-Ib-cr* and extended-spectrum beta-lactamase gene *bla*CTX-M-24 co-located on the same plasmid in one *Escherichia coli* strain from China. J Antimicrob Chemother.

[55] Liu BT, Liao XP, Yue L, Chen XY, Li L, Yang SS, Sun J, Zhang S, Liao SD, Liu YH (2013). Prevalence of beta-lactamase and 16S rRNA methylase genes among clinical *Escherichia coli* isolates carrying plasmid-mediated quinolone resistance genes from animals. Microb Drug Resist.

[56] Yang L, Yang L, Lu DH, Zhang WH, Ren SQ, Liu YH, Zeng ZL, Jiang HX (2015). Co-prevalance of PMQR and 16S rRNA methylase genes in clinical *Escherichia coli* isolates with high diversity of CTX-M from diseased farmed pigeons. Vet Microbiol.

[57] Fang L, Li X, Li L, Li S, Liao X, Sun J, Liu Y (2016). Co-spread of metal and antibiotic resistance within ST3-IncHI2 plasmids from *E. coli* isolates of food-producing animals. Sci Rep.

[58] Wong MH, Chen S (2013). First detection of *oqxAB* in *Salmonella* spp. isolated from food. Antimicrob Agents Chemother.

[59] Bai L, Zhao J, Gan X, Wang J, Zhang X, Cui S, Xia S, Hu Y, Yan S, Wang J, Li F, Fanning S, Xu J (2016). Emergence and diversity of *Salmonella enterica* serovar Indiana isolates with concurrent resistance to ciprofloxacin and cefotaxime from patients and food-producing animals in China. Antimicrob Agents Chemother.

[60] Wong MH, Yan M, Chan EW, Biao K, Chen S (2014). Emergence of clinical *Salmonella enterica* serovar typhimurium isolates with concurrent resistance to ciprofloxacin, ceftriaxone, and azithromycin. Antimicrob Agents Chemother.

[61] Zhang Z, Meng X, Wang Y, Xia X, Wang X, Xi M, Meng J, Shi X, Wang D, Yang B (2014). Presence of *qnr*, *aac(6′)-Ib*, *qepA*, *oqxAB*, and mutations in gyrase and topoisomerase in nalidixic acid-resistant *Salmonella* isolates recovered from retail chicken carcasses. Foodborne Pathog Dis.

[62] Kao CY, Chen CA, Liu YF, Wu HM, Chiou CS, Yan JJ, Wu JJ (2017). Molecular characterization of antimicrobial susceptibility of *Salmonella* isolates: first identification of a plasmid carrying *qnrD* or *oqxAB* in Taiwan. J Microbiol Immunol Infect.

[63] Lin D, Chen K, Wai-Chi Chan E, Chen S (2015). Increasing prevalence of ciprofloxacin-resistant food-borne *Salmonella* strains harboring multiple PMQR elements but not target gene mutations. Sci Rep.

[64] Wang Y, Zhang A, Yang Y, Lei C, Jiang W, Liu B, Shi H, Kong L, Cheng G, Zhang X, Yang X, Wang H (2017). Emergence of *Salmonella enterica* serovar Indiana and California isolates with concurrent resistance to cefotaxime, amikacin and ciprofloxacin from chickens in China. Int J Food Microbiol.

[65] Li XP, Fang LX, Song JQ, Xia J, Huo W, Fang JT, Liao XP, Liu YH, Feng YJ, Sun J. Clonal spread of *mcr*-1 in PMQRcarrying ST34 *Salmonella* isolates from animals in China. Sci Rep. 2016;6. 10.1038/srep38511 PMC5137007 27917926

[66] Cao X, Xu X, Zhang Z, Shen H, Chen J, Zhang K (2014). Molecular characterization of clinical multidrug-resistant *Klebsiella pneumoniae* isolates. Ann Clin Microb Anti.

[67] Yang HY, Nam YS, Lee HJ (2014). Prevalence of plasmid-mediated quinolone resistance genes among ciprofloxacin-nonsusceptible *Escherichia coli* and *Klebsiella pneumoniae* isolated from blood cultures in Korea. Can J Infect Dis Med Microbiol.

[68] Cheng L, Cao XL, Zhang ZF, Ning MZ, Xu XJ, Zhou W, Chen JH, Zhang JH, Shen H, Zhang K (2016). Clonal dissemination of KPC-2 producing *Klebsiella pneumoniae* ST11 clone with high prevalence of *oqxAB* and *rmtB* in a tertiary hospital in China: results from a 3-year period. Ann Clin Microbiol Antimicrob.

[69] Goudarzi M, Azad M, Seyedjavadi SS (2015). Prevalence of plasmid-mediated quinolone resistance determinants and OqxAB efflux pumps among extended-spectrum beta-lactamase producing *Klebsiella pneumoniae* isolated from patients with nosocomial urinary tract infection in Tehran, Iran. Scientifica.

[70] Domokos J, Kristof K, Szabo D (2016). Plasmid-mediated quinolone resistance among extended-Spectrum Beta-lactamase producing *Enterobacteriaceae* from bloodstream infections. Acta Microbiol Immunol Hung.

[71] Ferjani S, Saidani M, Amine FS, Boutiba-Ben BI (2015). Prevalence and characterization of plasmid-mediated quinolone resistance genes in extended-spectrum beta-lactamase-producing *Enterobacteriaceae* in a Tunisian hospital. Microb Drug Resist.

[72] Rafiq Z, Sam N, Vaidyanathan R (2016). Whole genome sequence of *Klebsiella pneumoniae* U25, a hypermucoviscous, multidrug resistant, biofilm producing isolate from India. Mem Inst Oswaldo Cruz.

[73] Abderrahim A, Djahmi N, Pujol C, Nedjai S, Bentakouk MC, Kirane-Gacemi D, Dekhil M, Sotto A, Lavigne J-P, Pantel A (2017). First case of NDM-1-producing *Klebsiella pneumoniae* in Annaba University hospital, Algeria. Microb Drug Resist (Larchmont, N Y).

[74] Zhong X, Xu H, Chen D, Zhou H, Hu X, Cheng G (2014). First emergence of *acrAB* and *oqxAB* mediated tigecycline resistance in clinical isolates of *Klebsiella pneumoniae* pre-dating the use of tigecycline in a Chinese hospital. PLoS One.

[75] Keeney D, Ruzin A, McAleese F, Murphy E, Bradford PA (2008). MarA-mediated overexpression of the AcrAB efflux pump results in decreased susceptibility to tigecycline in *Escherichia coli*. J Antimicrob Chemother.

[76] Keeney D, Ruzin A, Bradford PA (2007). RamA, a transcriptional regulator, and AcrAB, an RND-type efflux pump, are associated with decreased susceptibility to tigecycline in *Enterobacter cloacae*. Microb Drug Resist.

[77] Ruzin A, Keeney D, Bradford PA (2007). AdeABC multidrug efflux pump is associated with decreased susceptibility to tigecycline in *Acinetobacter calcoaceticus*-*Acinetobacter baumannii* complex. J Antimicrob Chemother.

[78] Dean CR, Visalli MA, Projan SJ, Sum P-E, Bradford PA (2003). Efflux-mediated resistance to tigecycline (GAR-936) in *Pseudomonas aeruginosa* PAO1. Antimicrob Agents Chemother.

[79] Chen Y, Hu DX, Zhang QJ, Liao XP, Liu YH, Sun J. Efflux pump overexpression contributes to tigecycline heteroresistance in *Salmonella enterica* serovar typhimurium. Front Cell Infect Mi. 2017;7. 10.3389/fcimb.2017.00037 PMC5313504 28261566

[80] Veleba M, De Majumdar S, Hornsey M, Woodford N, Schneiders T (2013). Genetic characterization of tigecycline resistance in clinical isolates of *Enterobacter cloacae* and *Enterobacter aerogenes*. J Antimicrob Chemother.

[81] Lin Y-T, Huang Y-W, Huang H-H, Yang T-C, Wang F-D, Fung C-P (2016). *In vivo* evolution of tigecycline-non-susceptible *Klebsiella pneumoniae* strains in patients: relationship between virulence and resistance. Int Antimicrob Ag.

[82] Juan C-H, Huang Y-W, Lin Y-T, Yang T-C, Wang F-D (2016). Risk factors, outcomes, and mechanisms of tigecycline-nonsusceptible *Klebsiella pneumoniae* bacteremia. Antimicrob Agents Chemother.

[83] Fransen F, Melchers MJ, Meletiadis J, Mouton JW (2016). Pharmacodynamics and differential activity of nitrofurantoin against ESBL-positive pathogens involved in urinary tract infections. J Antimicrob Chemother.

[84] Sandegren L, Lindqvist A, Kahlmeter G, Andersson DI (2008). Nitrofurantoin resistance mechanism and fitness cost in *Escherichia coli*. J Antimicrob Chemother.

[85] Ho PL, Ng KY, Lo WU, Law PY, Lai ELY, Wang Y, Chow KH (2016). Plasmid-mediated *oqxAB* is an important mechanism for nitrofurantoin resistance in *Escherichia coli*. Antimicrob Agents Chemother.

